# Multicomponent Non-Woven Fibrous Mats with Balanced Processing and Functional Properties

**DOI:** 10.3390/polym12091911

**Published:** 2020-08-25

**Authors:** Tatiana S. Demina, Anastasia S. Kuryanova, Polina Y. Bikmulina, Nadejda A. Aksenova, Yuri M. Efremov, Zulfar I. Khaibullin, Pavel L. Ivanov, Nastasia V. Kosheleva, Peter S. Timashev, Tatiana A. Akopova

**Affiliations:** 1 Enikolopov Institute of Synthetic Polymeric Materials, Russian Academy of Sciences (ISPM RAS), 70 Profsoyuznaya st., 117393 Moscow, Russia; khaibullin.zulfar@yandex.ru (Z.I.K.); ivanovpl@inbox.ru (P.L.I.); akopova@ispm.ru (T.A.A.); 2 Institute for Regenerative Medicine, Sechenov University, 8-2 Trubetskaya st., 119991 Moscow, Russia; kuryanovaanastasi@gmail.com (A.S.K.); polina_bikmulina@mail.ru (P.Y.B.); naksenova@mail.ru (N.A.A.); yu.efremov@gmail.com (Y.M.E.); timashev.peter@gmail.com (P.S.T.); 3 Semenov Institute of Chemical Physics, Russian Academy of Sciences, 4 Kosygina st., 119991 Moscow, Russia; 4 Faculty of Biology, Lomonosov Moscow State University, 12-1, Leninskie Gory, 119234 Moscow, Russia; n_kosheleva@mail.ru; 5 FSBSI “Institute of General Pathology and Pathophysiology”, 8, Baltiyskaya st., 125315 Moscow, Russia; 6 Chemistry Department, Lomonosov Moscow State University, 1-3 Leninskiye Gory, 119991 Moscow, Russia

**Keywords:** polyesters, gelatin, chitosan, solvent-free co-extrusion, non-woven mats, electrospinning, cytocompatibility

## Abstract

The mimicking of the architectonics of native tissue, biodegradable non-woven fibrous mats is one of the most promising forms of scaffolding for tissue engineering. The key properties needed for their successful application in vivo, such as biodegradability, biocompatibility, morphology, mechanical properties, etc., rely on their composition and appropriate 3D structure. A multicomponent system based on biodegradable synthetic (polycaprolactone, oligo-/polylactide) and natural (chitosan, gelatin) polymers, providing the desired processing characteristics and functionality to non-woven mats fabricated via the electrospinning technique, was developed. The solid-state reactive blending of these components provided a one-step synthesis of amphiphilic graft copolymer with an ability to form stable ultra-fine dispersions in chlorinated solvents, which could be successfully used as casting solvents for the electrospinning technique. The synthesized graft copolymer was analyzed with the aim of fractional analysis, dynamic laser scattering, FTIR-spectroscopy and DSC. Casting solution characteristics, namely viscosity, surface tension, and electroconductivity, as well as electrospinning parameters, were studied and optimized. The morphology, chemical structure of the surface layer, mechanical properties and cytocompatibility were analyzed to confirm the appropriate functionality of the formed fibrous materials as scaffolds for tissue engineering.

## 1. Introduction

The development of advanced functional materials possessing a number of specific features and properties is, nowadays, one of the most important tasks of materials science. Polymeric scaffolds, i.e., biodegradable temporary supports for cell adhesion/growth, are one of key component of successfully bringing the idea of tissue engineering into clinical practice [1,2]. Due to a wide range of criteria the scaffolds should meet, this mission is still unaccomplished. The complexity of this task from a material science point of view could be divided into two main problems: (1) the reproducibility and flexibility of scaffold processing in terms of the needs of specific patients, and (2) the ability of the final material to fulfill all requirements the scaffold should satisfy. Currently, the fabrication of scaffolds for real clinical applications need to be developed through industry [3]. From this point of view, the development of new scaffolds should be carried out by keeping in mind their manufacturing process.

There are several leading material processing technologies widely accepted for scaffold fabrication, such as additive technologies (3D printing, stereolithography, etc.) and electrospinning. The latter comprises the productivity, flexibility and suitable topography of fabricated porous non-woven fibrous scaffolds enabling tissue vascularization [4,5]. This technology allows varying material architectonics and is rather universal in terms of the nature of the processed polymers [6,7,8,9]. However, there is still no “ideal” polymer that is able to provide fabricated scaffolds of necessary functionality.

Synthetic (oligo-/polyesters) and natural (chitosan/gelatin) components possess a range of specific features promoting their application for the development of various materials for tissue engineering [10]. The first ones provide good mechanical properties and processability into complex 3D structures via a range of technological approaches, while natural ones possess biocompatibility and an affinity to native extracellular structures. Since the properties of these components complement each other, their combination is one of the popular topics of biomaterial science. The majority of approaches rely on the fabrication of composite materials, where polysaccharides/proteins are used as bioactive coatings or fillers [11,12,13]. This summons a lot of technological problems due to the low compatibility of these components. The electrospinning procedure of hydrochloride chitosan salt suspensions in polylactide-co-polycaprolactone copolymer was used for the fabrication of composite materials, but it required a post-treatment of fibrous materials to stabilize the chitosan within the fibers [7]. The fabrication of composite chitosan/polylactide fibrous materials is also possible using harsh solvents [14]. Therefore, the development of composite materials does not fit fully to the strategy of the clinical translation of regenerative medicine. The combination of the advantages of different polymers in a frame of one macromolecule via copolymerization is another approach that could be interesting in terms of both processability and final material properties. The same problem of component incompatibility arises when the combination is carried out on a macromolecular level, i.e., through the modification of chemical structures via copolymer synthesis. Traditional solvent- or melt-based synthesis routes are obstructed by either the inability of natural polymers to melt or the need to use harsh and toxic solvents during multi-step synthesis [15,16]. Mechanochemistry is an effective and eco-friendly approach to a combination of different macromolecules. This synthetic route relies on the activation of solid substances and their intensive intermixing by applying shear deformation [17,18]. Mechanochemistry was originally widely used for metals and inorganic components, but with the evolution of solid-state reactive extrusion, it is becoming a one of the hotspots in the chemical engineering of organic compounds [19]. A special benefit of this technology relies on the possibility of processing multicomponent blends and their effective combination via both intramolecular interactions and covalent bonds.

This work aims to develop a new multicomponent system based on synthetic and natural macromolecules via the mechanochemical approach, in order to evaluate its processability in non-woven mats through the electrospinning technique, as well as the ability of these mats to serve as scaffolds for tissue engineering.

## 2. Materials and Methods 

Polylactide (PLA) (Natureworks 4043D, Minnetonka, MN, USA) with a molecular weight (Mw) of 100 kDa, polycaprolactone (PCL) with a Mw of 67 kDa, as well as gelatin (Chimmed, Russia), were used. Chitosan with a Mw of 80 kDa and a degree of deacetylation of 0.89 was synthesized in ISPM RAS as reported earlier [20]. Oligo (l-lactide) with a Mw of 5 kDa was synthesized from l-lactic acids (Panreac, Barcelona, Spain). A multicomponent graft copolymer-based system was obtained via the mechanochemical approach using a pilot co-rotating twin-screw extruder (ZE40×23D, Berstorff, Germany) specially designed for the powerful dispersion of solids [21]. As a first step, a mixture of chitosan and gelatin (50/50 *w*/*w*) was co-extruded at 60 °C for 15 min at 60 rpm. Then, this mixture was co-extruded with oligo (l-lactide) for 10 min at 60 °C at the same rpm to give a 0.25 molar ratio per chitosan unit. These conditions were previously used to graft oligolactide fragments onto chitosan and to provide an amphiphilicity and better compatibility with polyesters [22]. The obtained chitosan/oligolactide/gelatin three-component blend was marked as COG. Afterwards, polylactide and polycaprolactone were co-extruded with the COG system at 30 °C for 15 min at 100 rpm. The ratio between synthetic (oligo-/polylactide, polycaprolactone) and natural (chitosan, gelatin) components in the final blend was 60/40 *w*/*w*, which was previously found to be optimal for the efficient mechanochemical treatment of the similar systems [23]. The target system consisted of polycaprolactone/polylactide/chitosan/oligolactide/gelatin, marked as PPCOG, and had a 18/37/20/5/20 weight ratio.

As a first step, we evaluated the effectiveness of oligolactide grafting onto chitosan within the COG mixture. Therefore, the COG sample probe (1.5 g) was dispersed in 20 mL of acetone for 2 h at room temperature (RT) under magnetic stirring. After the dissolution of unreacted oligolactide, the insoluble fraction was collected by filtration, washed several times on paper filter with acetone and dried in a vacuum oven. The grafting percentage was calculated as the percentage between the bonded oligolactide to the total amount of natural components. The fractionation of the prepared PPCOG copolymer in acidic medium was performed as follows: a portion of ~1 g taken from the sample was immersed in 75 mL of 2% acetic acid. After stirring for about 24 h at RT, insoluble fractions were collected by filtration, washed on the filter with distilled water to reach a neutral pH and freeze dried. The solids contained in the filtrate were precipitated by adding an equal amount of 5% ammonia aqueous solution, separated from the solutions by centrifugation, washed with water to reach neutral pH and freeze dried.

The solubility characteristics of the PPCOG copolymer dispersions in CH_2_Cl_2_ (0.2 mg/mL) were assessed by dynamic laser scattering (DLS) using a Zetatrac particle size analyzer (Microtrac, Inc., Montgomeryville, PA, USA) and the Microtrac application software program (V.10.5.3).

FTIR analysis of the initial components and the synthesized PPCOG system was carried out using a Spectrum Two FT-IR Spectrometer (PerkinElmer, Waltham, MA, USA) in Attenuated Total Reflectance (ATR) mode. The spectrometer features were as follows: high-performance, room-temperature LiTaO_3_ MIR detector, standard optical system with KBr windows for data collection over a spectral range of 8300–350 cm^−1^ at a resolution of 0.5 cm^−1^. All spectra were initially collected in ATR mode and converted into IR transmittance mode. The spectra of non-modified chitosan and its copolymers were normalized using the intensity of C–O stretching vibrations of a pyranose cycle band (1081 cm^−1^) as the internal standard.

Differential scanning calorimetry (DSC) measurements were performed using an STA 6000 simultaneous thermal analyzer (PerkinElmer, Waltham, MA, USA). Samples for DSC experiments (about 10 mg) were encapsulated in standard PerkinElmer aluminum pans and heated in a nitrogen medium at a gas flow rate of 40 mL/min and a linear heating rate of 10 °C/min. DSC thermograms and thermal properties obtained from non-isothermal DSC second heating run are shown as Figure S1 and Table S1 in the Supplementary Materials.

The viscosity (η) of the 1 wt % PLA and PPCOG solutions was measured using an electromagnetically spinning viscometer, EMS-1000 (Kyoto Electronics Manufacturing, Japan), at 25 °C, after a rest time of 3~5 min, applying a shear rate of 400 s^−1^. Electrical conductivity was evaluated using an Expert-002 conductometer (Volta, Russia), while surface tension (σ) was evaluated with a Du-Nui tensometer with a platinum ring detachment.

Non-woven fibrous scaffolds were fabricated from solutions of PLA or PPCOG in chloroform using ESR100D NanoNC (Seoul, South Korea). All processing parameters were preliminary optimized to obtain defect-free fibrous materials with a thickness of 100–120 μm. The distance between the needle tip and the steel plate support (30 × 30 cm), a conductive static collector, was 18–22 cm. Other optimized parameters of the electrospinning procedure are given in Table 1.

The morphology of the non-woven mats was studied by SEM using PhenomProX (ThermoFisher Scientific, Waltham, MA, USA) at 10–15 kV. The average fiber diameter and its distribution were calculated by an analysis of the SEM images using ImageJ software (version 1.52).

The mechanical properties of the PLA and PPCOG non-woven mat samples were tested in dry and wet states using Mach-1 v500csst Micromechanical Testing System (Biomomentum Inc., Laval, QC, Canada). The testing of the samples in wet mode was realized after their incubation in mQ water for 2 days during constant lateral stirring at 200–300 rpm at RT. The tensile strength, elongation at break and modulus of elasticity (Young’s modulus) were measured during the uniaxial tension of at least three dog bone-shaped samples (15 × 5 mm gauge) and reported as the mean ± standard deviation values.

The surface chemical composition of PPCOG non-woven mats was evaluated using fluorescent microscopy after staining with fluorescein isothiocyanate (FITC) (Sigma-Aldrich, St. Louis, MO, USA), a fluorescein dye selective toward chitosan amino groups and proteins. FITC staining was realized according to procedure published earlier [24]. The wettability of PLA and PPCOG fibrous mats was evaluated via measurements of the contact angles of sessile drops of distilled water (mQ) with the Acam-MSC01 (Apex Instruments, Kolkata, India). The surface contact angle values reported were the average of at least three measurements made on different areas of the fibrous membranes within 35 min after a water drop set.

The biocompatibility of the fibrous mats was analyzed using human mesenchymal stromal cells (MSCs) isolated from gingival mucosa, as described in [25]. The MSC cultural media consisted of Dulbecco’s Modified Eagle’s Medium (DMEM)/F12 (1:1, Biolot, St. Petersburg, Russia) supplemented with 10% fetal calf serum (HyClone, Logan, UT, USA), l-glutamine (5 mg/mL, Gibco, Gaithersburg, MD, USA), insulin–transferrin–sodium selenite (1:100, Biolot, St. Petersburg, Russia), bFGF (20 ng/mL, ProSpec, Rehovot, Israel), and gentamycin (50 μg/mL, Paneco, Moscow, Russia). Cell morphology and immunophenotype were routinely checked with a phase-contrast microscope Primovert (Carl Zeiss, München, Germany) and microfluidic cell sorter Sony SH800 (Sony Biotechnology, Bothell, WA, USA), respectively. The following human antibodies were used for flow cytometric MSC immunophenotyping: HLA-DR, CD14, CD19, CD34, CD45, CD90, CD73, CD105, CD29, and CD44, all conjugated with phycoerythrin (PE) and obtained from Miltenyi Biotec (Bergisch Gladbach, Germany). Each sample included 20,000 events, and 3 different cell population samples from passage 4 (p4) were tested.

For the assessment of the non-woven mats’ cytotoxicity, we used AlamarBlue cell viability reagent (Invitrogen, Waltham, MA, USA). Briefly, extracts of the PLA and PPCOG samples were prepared via incubation of a 1 cm^2^ piece in 1 mL of a culture media for 24 h at 37 °C each. Serial dilutions of the PLA extract were performed as a negative non-toxic control and sodium dodecyl sulphate (SDS) dilutions as a positive control. Extracts and SDS were added in triplicate to MSCs seeded in 96-well plates. Cells were incubated for 24 h at 37 °C in 5% CO_2_. For the AlamarBlue cell viability assay, cell media were replaced with a reagent solution according to the manufacturer’s protocol, and were incubated for the next 2 h. The fluorescence of the viable cells was quantified using a spectrofluorometer, Victor Nivo (PerkinElmer, Waltham, MA, USA), at a 530-nm excitation wavelength and a 590-nm emission wavelength.

To confirm the results obtained with the AlamarBlue assay, we utilized the Quant-iT PicoGreen kit (Invitrogen, Waltham, MA, USA) for the quantification of the DNA amount in the same samples. Afterwards, the AlamarBlue assay plates underwent 3 freeze–thaw cycles (30 min each) to destroy cell membranes and release intracellular DNA. Then, all operations were performed following using standard protocols, and DNA fluorescence intensity was detected using a spectrofluorometer (Victor Nivo) at a 480-nm excitation wavelength and a 520-nm emission wavelength.

The visualization of the cell viability and morphology after growth on the PLA and PPCOG mats was achieved with a Live/Dead assay. Live cells were stained with Calcein-AM (Sigma -Aldrich, St. Louis, MO, USA), dead cells with Propidium Iodide (PI, Thermofisher, Waltham, MA, USA), and additional nuclei staining was performed with Hoechst 33258 (Thermofisher, Waltham, MA, USA). The images of stained samples were obtained using LSM 880 with Airyscan (Zeiss, Oberkochen, Germany) and a 352-nm laser, a 488-nm argon laser and a 561-nm laser to visualize the Hoechst 33258, Calcein-AM, and PI signals, respectively. The visualization was performed in the z-stacking mode.

## 3. Results

### 3.1. Copolymer Synthesis and Characterization

#### 3.1.1. Fractional Analysis

The oligolactide grafting degree within the preliminary COG blend was 14.7 wt %. The grafting degree of oligo-/polylactide/polycaprolactone fragments onto chitosan/gelatin within the final PPCOG sample was difficult to estimate due to the insolubility of polyesters with a high Mw in acetone and the tendency of the PPCOG blend to form ultra-fine stable colloidal systems in CH_2_Cl_2_/CHCl_3_, which are traditionally used for PLA and PCL dissolution. The results of the step-by-step fractional analysis of PPCOG in mQ and 2% AcOH (good solvents to gelatin and chitosan, respectively) are shown in Table 2.

#### 3.1.2. Dynamic Laser Scattering

**Figure 1:**
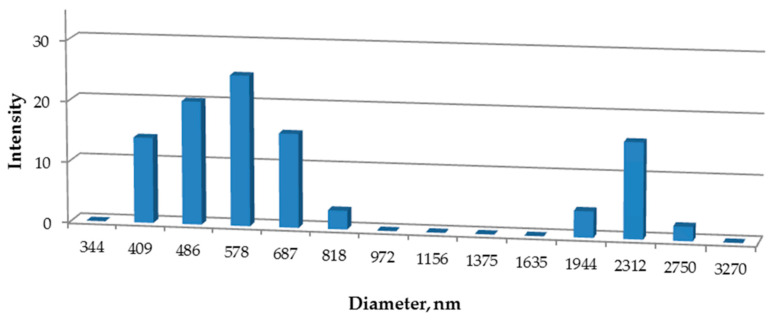
Size distribution of the 0.2 wt % PPCOG dispersions in CHCl_3_.

The ability of the PPCOG to form stable ultra-fine colloidal dispersions was estimated via measurements of the size and size distribution of the formed macromolecular associates in a 0.2 wt % solution of CHCl_3_. Figure 1 shows a histogram of the macromolecular associate sizes measured via DLS analysis.

#### 3.1.3. FTIR-Spectroscopy

**Figure 2:**
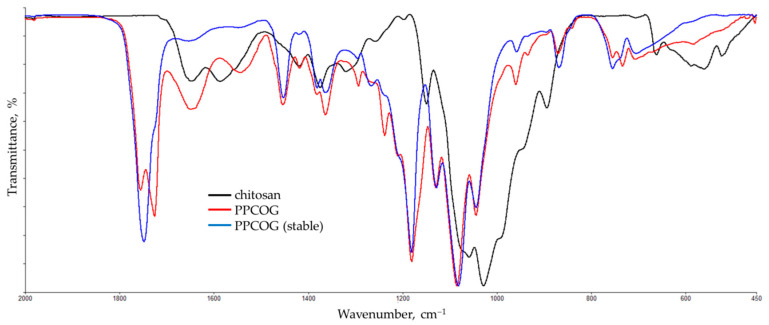
IR spectra of non-modified chitosan and PPCOG system (a whole sample and stable in chloroform fraction).

In order to reveal the changes in the structure of the blend components, the FTIR spectra of non-modified chitosan and the PPCOG system were recorded. All spectra presented in Figure 2 were normalized using the intensity of C–O stretching vibrations of a pyranose cycle band (1081 cm^−1^) as the internal standard [26]. The band of the deformation vibrations of NH_2_ groups at 1590 cm^−1^, as well as the Amid-I and Amide-II vibrations of the residual acetamide groups of chitin at 1650 and 1540 cm^−1^, respectively, were observed in the spectrum of the initial chitosan. The typical IR bands of the ester groups are found between 1760 and 1730 cm^−1^ in the carbonyl stretching vibrations as well as at 1186/1135/1093 cm^−1^ in the –C(O)–O– deformation.

### 3.2. Characterization of PLA and PPCOG Non-Woven Mats

#### 3.2.1. Morphology of Non-Woven Mats

The main polymer solution characteristics affecting the electrospinning procedure, such as viscosity, conductivity and surface tension, were measured and are summarized in Table 3.

**Figure 3:**
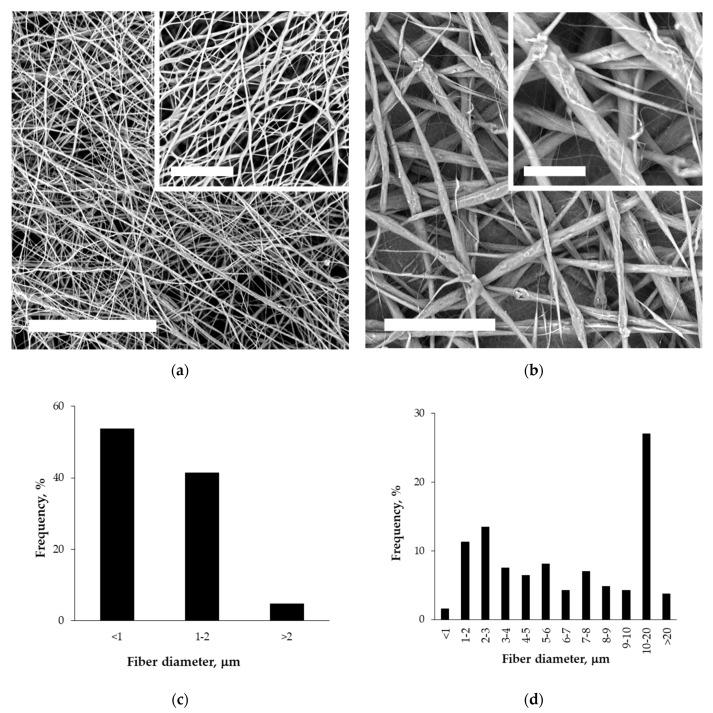
SEM micrographs (**a**,**b**) and histograms (**c**,**d**) of the fiber diameter distribution for non-woven mats fabricated from PLA (**a**,**c**) and PPCOG (**b**,**d**). The scale bar is 100 μm for the survey images and 30 μm for the inserted ones.

SEM micrographs of PLA and PPCOG non-woven mats and histograms of the fiber size distribution calculated from these micrographs are shown in Figure 3.

#### 3.2.2. Surface Properties of Non-Woven Mats

**Figure 4:**
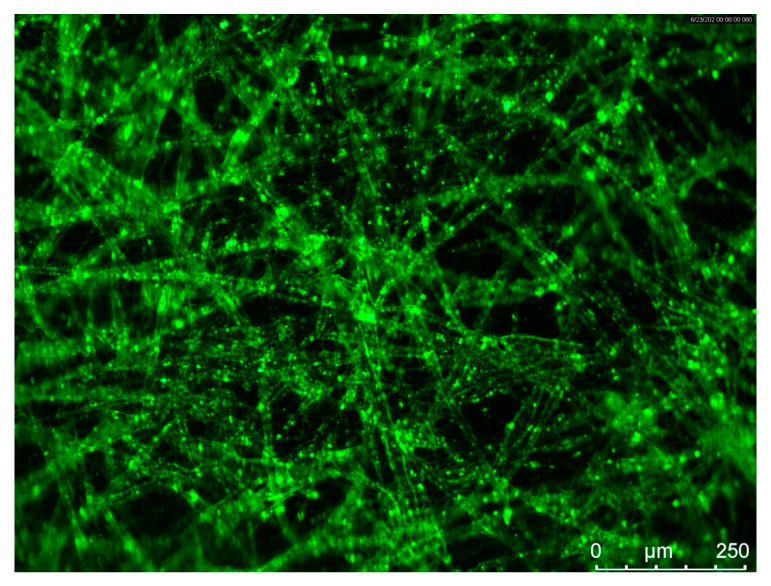
Fluorescent micrograph of PPCOG sample after staining with fluorescein isothiocyanate (FITC).

The surface features of PLA and PPCOG mats were evaluated in terms of their surface chemical structure and wettability. A fluorescent micrograph of the PPCOG sample after staining with FITC, the fluorescein dye selective to chitosan amino groups and proteins, is shown in Figure 4.

**Figure 5:**
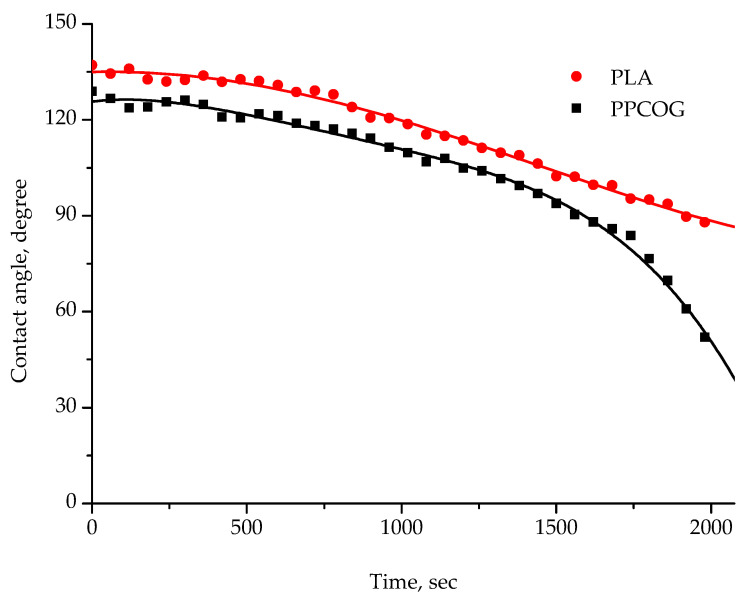
Dependence of contact angle of wettability of PLA and PPCOG mats on time.

The initial contact angles of wettability and their time evolution are shown in Figure 5. The PLA fibrous material was more hydrophobic than the PPCOG one at the beginning of the measurements. Moreover, water showed only a limited tendency to spread on the PLA sample, reaching the contact angle of 88° half an hour after a drop set, while the contact angle of wettability of PPCOG decreased to 52° (in 2.5-fold from initial value) within the same time.

#### 3.2.3. Mechanical Properties of Non-Woven Mats

**Figure 6:**
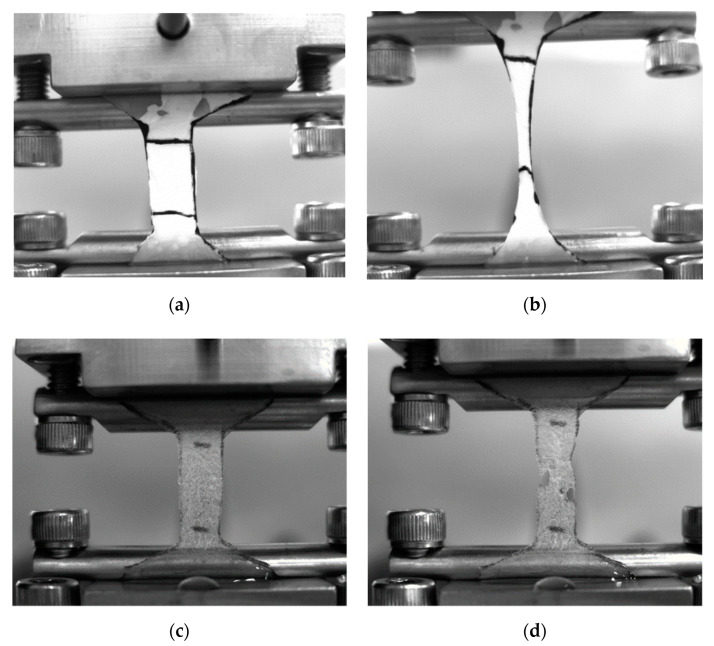
Photos of (**a**,**b**) PLA and (**c**,**d**) PPCOG fibrous mats in wet state (**a**,**c**) at the beginning and (**b**,**d**) at the end of the mechanical testing.

Keeping in mind the future application of the samples as scaffolds for cell cultures, both types of non-woven mats were tested in terms of their mechanical properties in dry and wet states. Macrophotos of the samples at the beginning and at the end of the deformation are shown in Figure 6, while the obtained results of the mechanical testing are summarized in Table 4.

#### 3.2.4. Biocompatibility of Non-Woven Fibrous Mats

**Figure 7:**
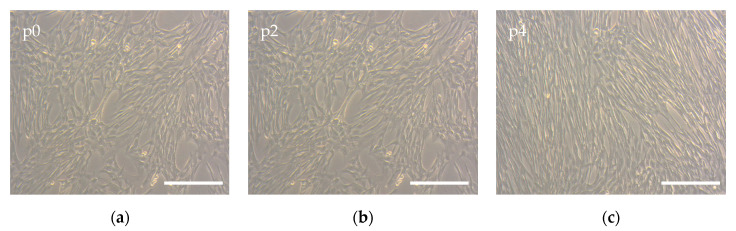
The morphology of the MSCs isolated from human gingiva: passage 0 (**a**) and passages 2 and 4 expanded in laboratory conditions (**b**,**c**), obtained by phase contrast microscope. The scale bar is 100 μm.

To correctly assess the effects of the non-woven polymeric mat’s properties on cell physiology, the phenotype of the primary MSCs was first checked. Cells grown in Petri dishes on the tissue culture polystyrene (TCPS) had a normal morphology, revealing a spindle-shaped form, which was maintained from cell isolation (p0), to the expanded cell culture used in the biocompatibility experiments (p4) (Figure 7). These observations were confirmed by flow cytometry analysis. As is shown in Table 5, the expression of all negative markers did not exceed 5% of the cell population, and positive markers were expressed in more than 95% of the population. More detailed data about specific marker expression can be found in the Supplementary Materials (Figure S2). Taken together, these data allow us to consider cells isolated from human gingiva as MSCs [27].

**Figure 8:**
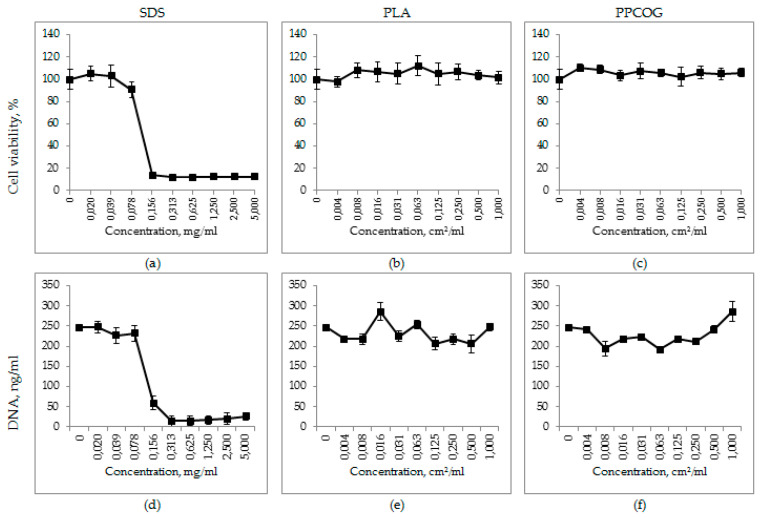
Relative cell viability (**a**–**c**) and DNA quantity (**d**–**f**) curves for sodium dodecyl sulphate (SDS) (**a**,**d**), PLA (**b**,**e**) and PPCOG (**c**,**f**). SDS is the positive control, and PLA is the negative control.

The AlamarBlue test of cells cultured both on PPCOG and PLA mats showed no harmful effects on the metabolic activity of the MSCs. Relative cell viability was around 100%, whereas cells exposed to SDS showed remarkable cell death (IC50 ≈ 0.2 mg/mL) (Figure 8a–c). The same patterns were presented by the DNA quantification test, where the DNA count in PPCOG and PLA samples varied from 200 to 300 ng/mL compared to 250 ng/mL in the control and 0–50 ng/mL DNA in samples exposed to a high SDS concentration (Figure 8d–f).

**Figure 9:**
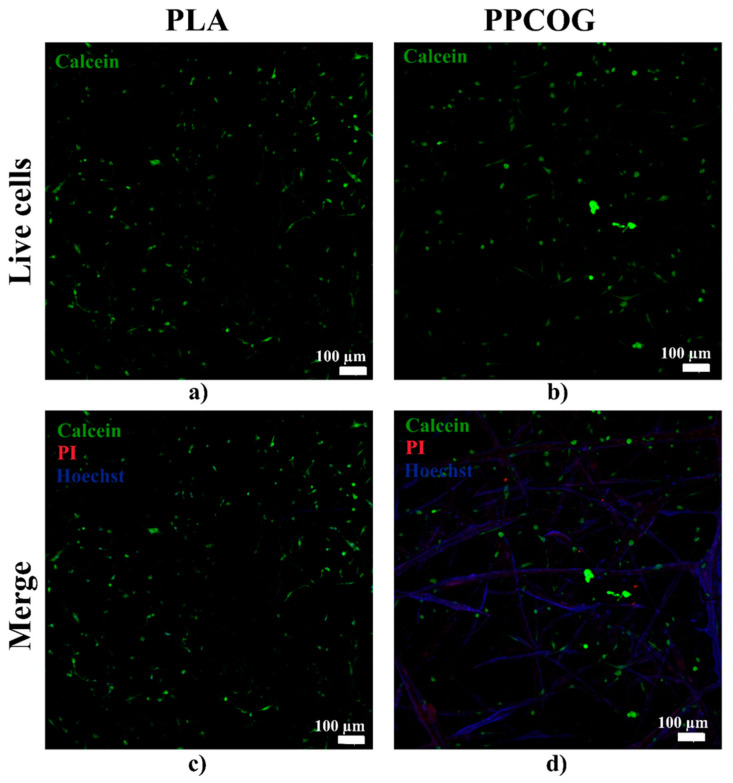

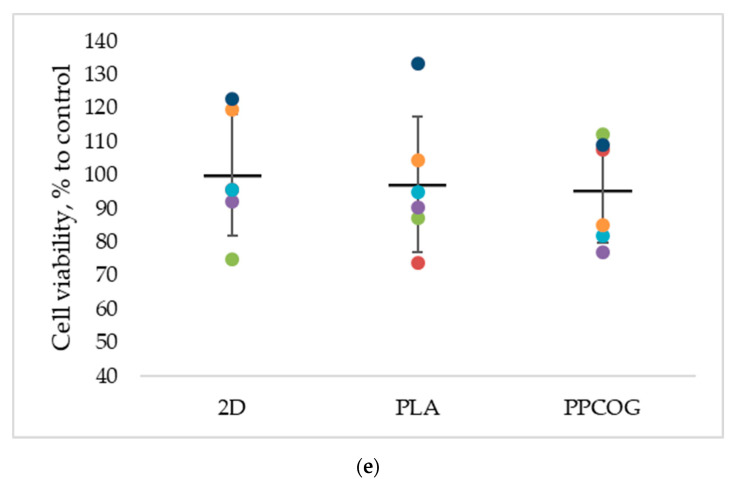
Live/Dead assay combined with nuclei staining for MSCs grown on PLA (**a**,**c**) or PPCOG (**b**,**d**). Live cells are stained with Calcein and shown as green (**a**,**b**), dead cells are stained with PI and shown red, and nuclei are stained with Hoechst and shown blue (**c**,**d**). Metabolic activity of the MSCs grown on tissue culture polystyrene (TCPS) (2D), PLA or PPCOG (**e**).

Live/Dead and additional nuclei staining also did not show any signs of contact cytotoxicity. After 7 days in culture, a high number of green viable cells can be observed on both types of the scaffold (Figure 9a,b). Only a few inclusions of the dead red cells were found (Figure 9c,d). Confocal images with bigger magnification are also given in the Supplementary section (Figure S3). MSCs attached to the PPCOG or PLA surface formed a perceptible amount of cell–cell contacts. However, cells cultivated on PPCOG were more spread out within the scaffold, while on the PLA they predominantly grew on the surface. Visually, it can be noted that there are a lower number of visible cells on the PPCOG. It also should be noticed that PPCOG revealed significant autofluorescence in blue and red channels (Figure 9d).

An AlamarBlue assay was additionally used to estimate the metabolic activity of the MSCs grown on the PPCOG surface after 3 days of cultivation. This assay involves resazurin, a redox indicator which becomes fluorescent after the transition to its reduced form, resorufin. Resazurin can be reduced by FMNH2, FADH2, NADH, NADPH, and cytochromes, which provide a general insight into the metabolic state of the cell. This test also depends on viable cell concentrations, and therefore can reflect the inhibition of cell proliferation or cell death. None of the significant alterations between cell viability and metabolic activity have been shown for the 2D control, PLA control and PPCOG, which confirms the previous data about the full biocompatibility of the polymer (Figure 9e).

## 4. Discussion

In spite of the fact that mechanochemistry allows us to effectively combine, within one macromolecule, such different components, in this research, we firstly grafted oligolactide fragments onto chitosan and gelatin to enhance their compatibility at the second synthesis stage with PLA and PCL [22]. Further processing of the prepared blend led to the fabrication of an amphiphilic polyester-based PPCOG system containing 40 wt % of natural components, which are normally soluble in aqueous acetic acid, while the results (Table 2) show that only 18.7 wt % of the composition are able to dissolve in aqueous solvents.

The decreased solubility in aqueous media was compensated by the ability to form ultra-fine dispersions within chlorinated solvents. This property is totally different from that which would be observed for a physical mixture of the parent materials. Obviously, the neat chitosan and gelatin would never dissociate in these solvents at all. According to DLS analysis, the employed conditions of their blending with polyesters result in a rather broad bimodal size distribution of the blend in CHCl_3_. The main part of the PPCOG in chloroform gave a colloidal solution with an approximate mean macromolecular associate size of 550 nm. It was previously shown by Correlo et al. that the melt-processing of chitosan and PLA mixture (at 50 wt % of chitosan) in an extruder did not change the initial size of chitosan particles (5 µm) [28]. On the contrary, the mechanochemical approach, which is based on the activation of solids and their intensive mixing during plastic deformation by applying high pressure and shear deformation below the melting temperatures of thermoplastic components, provides much more possibilities, up to and including the synthesis of copolymers [23,29]. The statistical nature of all possible routes of grafting predetermines a wide distribution of macromolecules in terms of their chemical structure, such as the number and length of grafted chains, and affects the behavior of the obtained blends in various solvents. Since bimodal distribution was observed due to the presence of macromolecular aggregates or microparticles, the fraction, which was stable in chloroform after keeping the dispersions for a week, was also investigated.

The analysis of DSC curves indicates the occurrence of the specific interaction of polyester chains with chitosan and gelatin moieties. The extrusion of polyesters in the presence of natural components results in the preparation of the PPCOG system, whose melting endotherm clearly differs from those of neat PLA and PCL (Figure S1). For instance, the blend endotherm comprises complementary peaks belonging to newly formed phases differing in terms of their thermodynamic stability [30]. For the PPCOG blend melted in DSC and then cooled at 10 °C min^−1^ rate, broad exothermic peaks were observed during the second DSC heating run in a temperature range close to the melting peaks of both pure polyesters, then shifting to a relatively lower (152 vs. 165 °C) and a relatively higher (62 vs. 60 °C) melting point than that of neat PLA and PCL, respectively. In the second heating run, the PLA homopolymer demonstrated a glass transition at 62 °C, but no melting peak was observed in contrast to the blend composition. This fact infers that natural components exert a clear nucleating effect on PLA. Similar observations were reported for PLA/chitosan blends (with a chitosan content of 40–60 wt %.) processed in an extruder at higher [28] and lower [23] temperatures in comparison with PLA’s melting point.

The FTIR spectra of non-modified chitosan and the PPCOG system were analyzed to reveal changes in the chemical structure of the components. The doublet of the bands at 1760 and 1730 cm^−1^ belongs to the C=O stretching vibrations in the ester group of PLA and PCL, respectively [31,32,33]. It should be noted that the relative intensity of PCL carbonyl bands was disproportionately increased; meanwhile, the content of PLA in the mixture was two times higher. This indicates conformational changes in the polyester macromolecules due to both physical and chemical intermolecular interactions between the mixture components, including natural fragments [34]. The shape of this doublet returns to normal in the spectrum of the fraction stabile in the chloroform (Figure 2), most likely caused by phase separation in the PLA/PCL mixtures when kept in a solvent. At the same time, part of the natural components aggregate and precipitate, which can be seen in the significant decrease in the intensity of the bands in the region of 1560–1650 cm^−1^, which are attributed to the amino and amido groups of chitosan and gelatin. The analysis of this region in the spectra of the reaction products also discovered the disappearance of the band of deformation vibrations of NH_2_, groups which can be seen at 1590 cm^−1^ in the spectrum of initial chitosan. Simultaneously, the intensities of the Amid-I and Amide-II vibrations of amide groups (Figure 2, see: peaks at 1650 and 1540 cm^−1^) noticeably increase, which indicates intensive grafting processes due to the aminolysis of ester bonds with the amino groups of chitosan and gelatin and the formation of graft copolymers with amide groups at branch points [35,36,37].

The copolymer formation opened a door to novel pathways of material fabrication, which are not allowed for native biopolymers. The grafting of oligo/polyester fragments onto a chitosan backbone was previously shown as a successful approach for the enhancement of material processability and properties [22,24,38,39,40]. The development of complex macromolecules comprising the benefits of different polymers has significant benefits in comparison with the fabrication of composite materials. It is easier from the point of view of material processing, since it requires a minimal number of technological steps and reduces the number of possible processing obstacles [9,11]. Moreover, a change in the chemical structure allows us to extend the number of technologies applied for material processing. For example, the fabricated PPCOG could be transformed into a material using both aprotic solvents and melt-based techniques, which are mostly unappropriated for non-modified polysaccharides or proteins.

The presence of natural components within PPCOG system led to significant differences in the properties of its solution in comparison with the solution of native PLA (see Table 3). The lower viscosity of the PPCOG sample was obviously caused by a lower amount of components soluble in chloroform (i.e., oligo/polyesters), but the possible decrease in their Mw due to aminolysis/alcoholysis reactions with functional groups of natural biopolymers could be a reason as well. On the other hand, the presence of natural components containing a variety of ionic groups (-COOH groups of aspartic and glutamic acid, -NH_2_ groups of chitosan, lysine, and hydroxylysine, and -NH-C(NH)-NH_2_ groups of arginine) led to an increase in the PPCOG solution’s conductivity in contrast to PLA’s. At the same time, the presence of chitosan/gelatin did not lead to notable changes in surface tension.

A difference in solution characteristics caused a need to optimize the electrospinning conditions for the fabrication of either PLA or PPCOG non-woven defect-free mats. As can be seen in Figure 3, the complex chemical composition of PPCOG led to the formation of fibers with a more complex topography in terms of fiber surface morphology and fiber size distribution. At the same time, blend fibers exhibited a homogeneous inner morphology, while fibers with similar weight ratios to PLA/PCL, manufactured through multilayer coextrusion, displayed a droplet-in-matrix morphology [41]. The optimal electrospinning conditions for PLA and PPCOG solutions were different due to the specific features of these macromolecules (cf. Table 1 and Table 3). The lower conductivity of the PLA solution was compensated by the higher voltage used for the mats’ fabrication, while the lower viscosity of PPCOG was equilibrated by the higher polymer concentration within the casting solution. The formed PPCOG mats consisted of fibers with a wider diameter distribution than PLA and the mean size of the copolymer fibers was significantly bigger than those in PLA: 7.6 ± 5.8 and 1.1 ± 0.4 μm, respectively. The variation in fiber diameter within PPCOG mats might be caused by an unstable polymer feed via the needle due to the aggregation of macromolecular associates within the concentrated copolymer colloidal solution. The presence of submicron aggregates could be a reason for the heterogeneous fluorescein emission coming from PPCOG mats stained with FITC (see Figure 4). This fluorescein dye is selective toward natural components only (i.e., chitosan/gelatin) and its spontaneous local concentration within PPCOG fiber’s spots could be related to the higher concentration of the chitosan/gelatin moieties in the form of macromolecular aggregates. The surface of FITC-labeled PPCOG mats showed a prominent emission in a green channel, confirming the presence and accessibility of biopolymers. The presence of natural macromolecules at the PPCOG surface led to its higher hydrophilicity in contrast to the PLA mat. The initial contact angle of wettability of PPCOG was 129 ± 1°, while its value for PLA was 137 ± 1° (Figure 5). Moreover, the long-term measurements of the contact angle of wettability showed the higher tendency of water to spread in copolymer mats. This feature could be caused by either the more complex topography of PPCOG mats or the presence of hydrophilic biopolymers on their surface, but both these fiber properties were in favor of the PPCOG sample in comparison with the PLA mat.

The accessibility of hydrophilic natural components also affected the mechanical behavior of the fibrous mats. As can be seen in Figure 6 and Table 4, the PLA-based mat showed classical deformation properties that were rather independent of sample state. On the contrary, the properties of the PPCOG fibrous mats in the wet state were significantly higher than those in the dry state. Although the addition of PCL to PLA at an optimal ratio (less than 20 wt %) usually improves the plastic properties and toughness of the materials obtained using different blending methods and processes, in particular by injection molding and hot pressing [42,43], the main influence is exerted by natural components. Generally, the presence of natural biopolymers decreased drastically (in approx. 10-times) in all evaluated mechanical characteristics of PPCOG fibrous mats in comparison with PLA samples. This result is expected, taking into account the high amount of chitosan/gelatin, which acted mainly as inert rigid filler in terms of the mechanical characteristics. The incubation of PPCOG mats in water before the testing obviously led to the swelling of the hydrophilic biopolymers and to the manifestation of their elastic properties. The mechanical properties of both types of fibrous mats are in the range of the mechanical properties of connective tissues [44]. Keeping in mind the intended application of the fibrous mats as scaffolds for cell cultures, we believe that the mechanical properties of the samples in the wet state should be considered as the functional ones.

A range of widely used cell-based in vitro methods confirmed the biocompatibility of the PPCOG mats. Neither quantitative (AlamarBlue and DNA count tests) nor qualitative (Live/Dead staining) assays showed any pronounced cytotoxic effects of the PPCOG mats. However, the MSC morphology was slightly affected during growth on both PLA and PPCOG non-woven mats. Such phenotype variety can be caused by the altered mechanical properties of the substrate [45,46]. Moreover, it has been shown that cells need a proper surface patterning for alignment, while relatively soft porous substrates often cause a round cell morphology [47,48]. Therefore, PPCOG performed as an adhesive and porous biomesh, which makes it an excellent model for 3D cell growth. The only issue obstructing the ease of PPCOG’s use is its intense autofluorescence. However, this problem can be solved by using more sensitive and selective fluorescent dyes.

## 5. Conclusions

Our multicomponent composition based on biodegradable synthetic (polycaprolactone, oligo-/polylactide) and natural (chitosan, gelatin) polymers was chosen to optimize the effectiveness of the mechanochemical approach to component combination, as well as the final features of the system in terms of processability via electrospinning and the wide range of functional characteristics of the developed fibrous material. The obtained blend demonstrated a tendency to form ultra-fine stable colloidal systems with a mean macromolecular associate size of 550 nm in CH_2_Cl_2_/CHCl_3_, which are traditionally used for PLA and PCL dissolution. The results of dynamic laser scattering, fractionation and FTIR analysis indicate the formation of graft copolymers in the course of the mechanochemical treatment of the reactive mixture due to the aminolysis of the ester bonds of polyesters with the amino groups of chitosan and gelatin. Non-woven fibrous scaffolds were fabricated from the copolymer solutions in chloroform at processing parameters optimized to obtain defect-free fibrous materials. The morphology, chemical structure of the surface layer, mechanical properties and cytocompatibility confirm the appropriate functionality of the formed fibrous materials as scaffolds for tissue engineering.

## Figures and Tables

**Table 1:** Electrospinning parameters for fabrication of polylactide (PLA) and polycaprolactone/polylactide/chitosan/oligolactide/gelatin (PPCOG) non-woven scaffolds.

Polymer Type	Concentration in Chloroform, wt %	Needle Type (Inner Diameter, mm)	Voltage, kV
PLA	15	23G (0.337)	23–26
PPCOG	25	21G (0.514)	13–17

**Table 2:** Fractional analysis of the PPCOG system in various solvents.

Sample	wt % of Fraction		
	Soluble in mQ (Enriched with Gelatin)	Soluble in 2% AcOH	Insoluble in Aqueous Medium
PPCOG	11.2	7.5	81.3

**Table 3:** Characteristics of the PLA and PPCOG solutions in chloroform.

Sample	η, mPa s	Conductivity, μS/cm	σ, mN/m
1% PLA	3.34 ± 0.02	0.010 ± 0.005	28.3 ± 0.3
1% PPCOG	2.23 ± 0.07	0.036 ± 0.003	27.6 ± 0.6

**Table 4:** Mechanical properties of PLA and PPCOG non-woven mats in dry and wet state.

Sample	PLA		PPCOG	
	Dry	Wet	Dry	Wet
Tensile strength, MPa	1.2 ± 0.7	1.4 ± 0.3	0.1 ± 0.01	0.3 ± 0.2
Elastic modulus, MPa	12 ± 8	8 ± 3	1.7 ± 0.6	6 ± 4
Elongation at break, %	90 ± 20	70 ± 10	7 ± 1	11 ± 4

**Table 5:** Immunophenotype of the p4 mesenchymal stromal cells (MSCs) isolated from human gingiva.

**Negative Markers**	**IgG1**	**CD14**	**CD19**	**CD34**	**CD45**
Expression, %	0.3 ± 0.1	0.6 ± 0.3	0.4 ± 0.1	1.8 ± 0.6	0.2 ± 0.1
**Positive Markers**	**CD29**	**CD44**	**CD90**	**CD73**	**CD105**
Expression, %	98.8 ± 0.7	99.96 ± 0.3	98.7 ± 0.4	99.9 ± 0.1	96.5 ± 1.2

## Supplementary Materials

The following are available online at https://www.mdpi.com/2073-4360/12/9/1911/s1, Figure S1: Second heating run DSC curves for neat PCL (1), neat PLA (2), stable fraction of PPCOG (3), and the whole PPCOG sample (4)., Figure S2: FACS histograms illustrating MSC passage 4 immunophenotype: negative markers (HLA-DR, CD14, CD19, CD34, CD45) and positive markers (CD90, CD73, CD105, CD29, CD44). Each specific protein is marked blue; IgG1 performed as the isotype control and is marked green. All of the presented markers (including IgG1) were conjugated with PE, Figure S3: Live/Dead assay combined with nuclei staining for MSCs grown on PLA (a,c) or PPCOG (b,d). Live cells are stained with Calcein and shown as green (a,b), dead cells are stained with PI and shown red, and nuclei are stained with Hoechst and shown blue (c,d). Table S1: Thermal properties obtained from non-isothermal DSC second heating at 10 °C min^−1^. The enthalpies of melting have been normalized by the weight fraction of the samples.
