# Supplementary material for: Multicomponent Non-Woven Fibrous Mats with Balanced Processing and Functional Properties
Source: Polymers (Basel). 2020 Aug 25;12(9):1911. doi: 10.3390/polym12091911 (PMC7563478; doi:10.3390/polym12091911)
Supplement: Supplementary file 1 [file polymers-12-01911-s001.pdf]

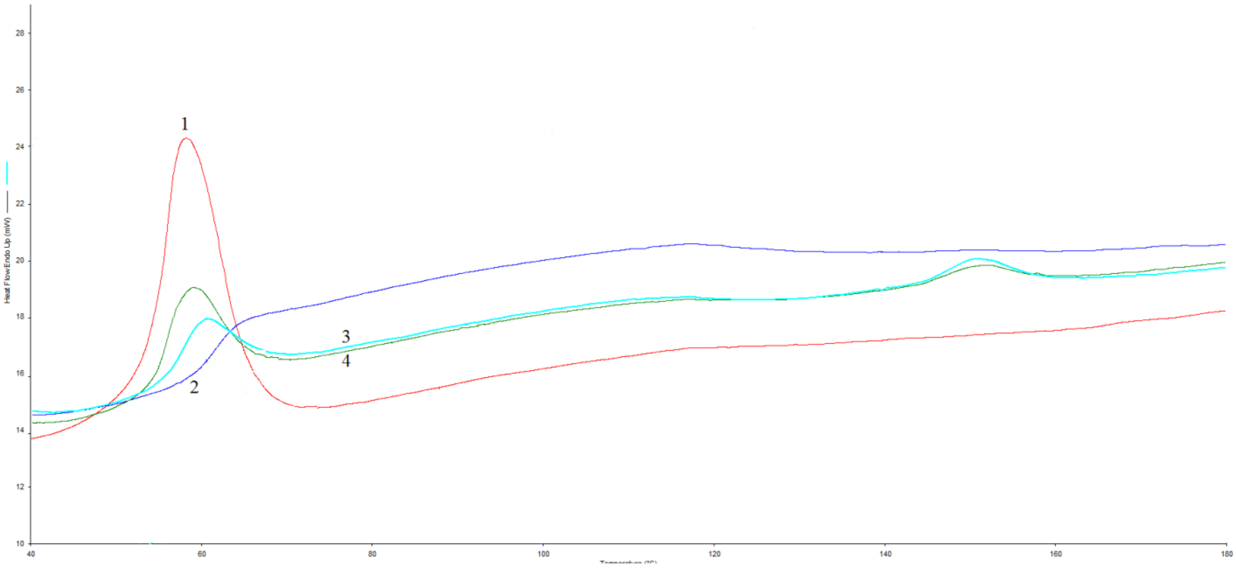

Figure S1. Second heating run DSC curves for neat PCL (1), neat PLA (2), stable fraction of PPCOG (3), and the whole PPCOG sample (4).

Table S1. Thermal properties obtained from non-isothermal DSC second heating at 10 °C min−1. The enthalpies of melting have been normalized by the weight fraction of the samples.

**Sample**	**PCL**		**PLA**	
	**T_m (°C)**	**ΔH_m(Jg^{-1})**	**T_m (°C)**	**ΔH_m (Jg^{-1})**
PLA	–	–	–	–
PCL	58.3	10.5	–	–
PPCOG (stable fraction)	58.9	3.6	151.7	0.8
PPCOG	60.6	2.1	150.8	1.1

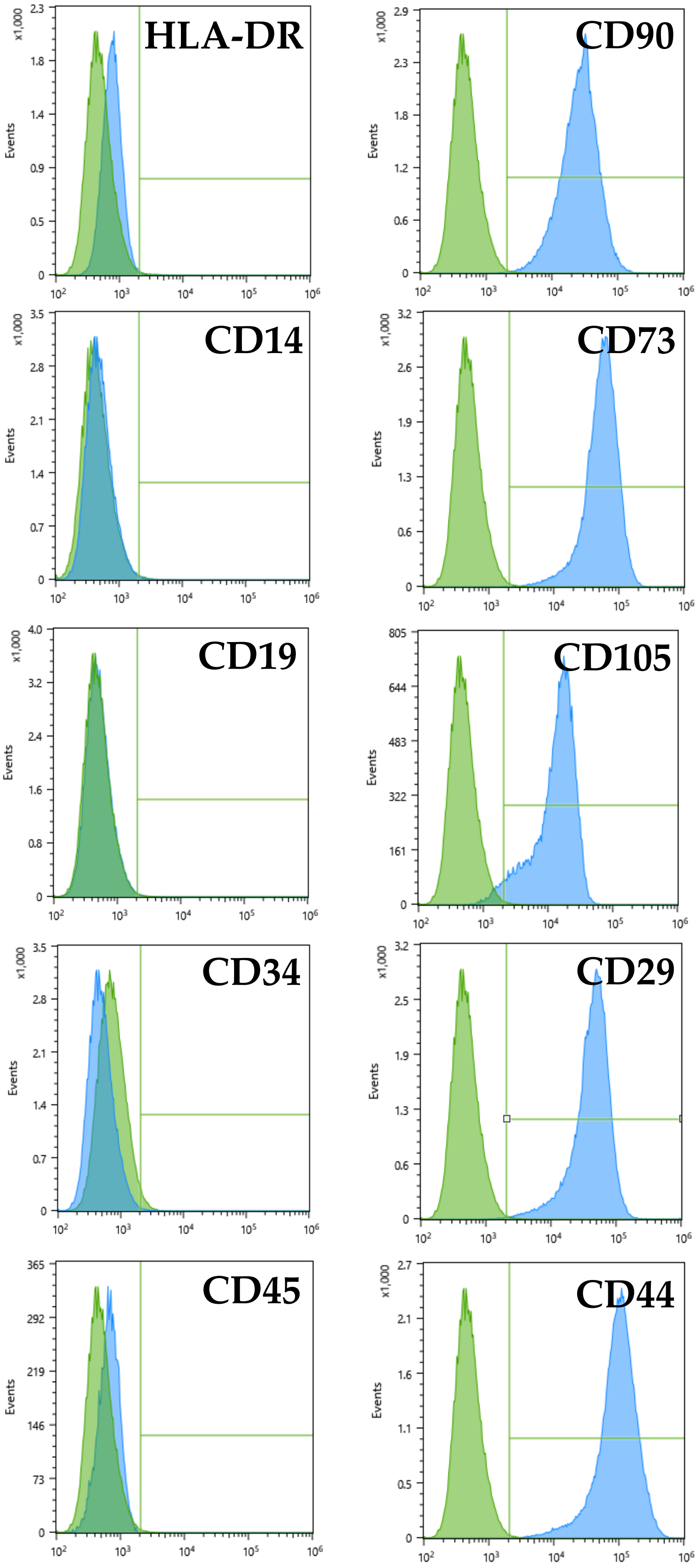

Figure S2. FACS histograms illustrating MSC passage 4 immunophenotype: negative markers (HLA-DR, CD14, CD19, CD34, CD45) and positive markers (CD90, CD73, CD105, CD29, CD44). Each specific protein is marked blue; IgG1 performed as the isotype control and is marked green. All of the presented markers (including IgG1) were conjugated with PE.

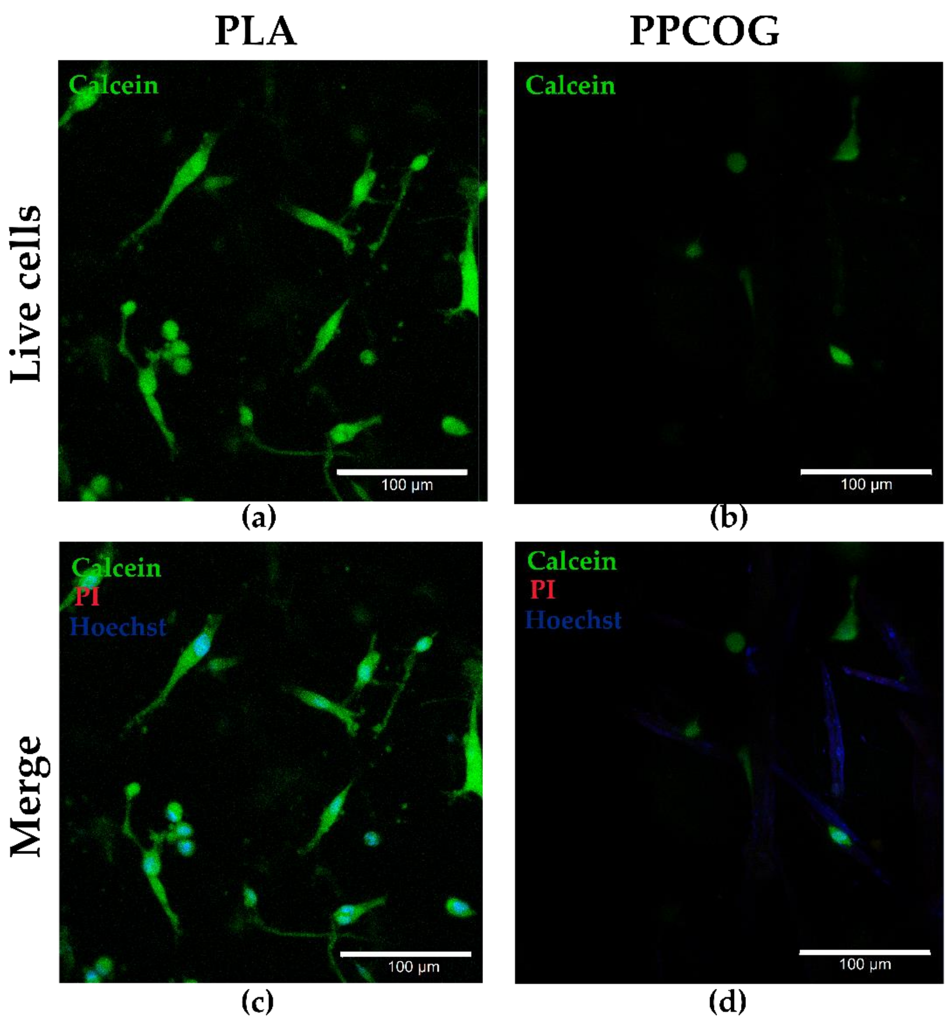

Figure S3. Live/Dead assay combined with nuclei staining for MSCs grown on PLA (**a, c**) or PPCOG (b, d). Live cells are stained with Calcein and shown as green (**a, b**), dead cells are stained with PI and shown red, and nuclei are stained with Hoechst and shown blue (**c, d**).
